# Vocal impact on quality of life of elderly female subjects

**DOI:** 10.1016/S1808-8694(15)31307-0

**Published:** 2015-10-20

**Authors:** Henrique Olival Costa, Cristiane Matias

**Affiliations:** ^1^ Otorhinolaryngologist, Head and Neck Surgeon, Ph.D. in Otorhinolaryngology, Joint Professor, Department of Otorhinolaryngology, Santa Casa de Sao Paulo (Coordinator of the Program of Post-graduation in Otorhinolaryngology, Santa Casa de Sao Paulo); ^2^ Speech Therapist, Master in Speech and Hearing Therapy, PUC-SP; Professor of Speech and Hearing Therapy, FAMERP

**Keywords:** voice, age, senior, quality, life

## Abstract

Although there are several investigations focusing the physiology and anatomy of voice and the senior's larynx, little has been produced to support the knowledge of the impact of vocal conditions on quality of life of this portion of the population.

**Aim:**

To verify the impact of voice on quality of the life of elderly women, using the questionnaires Short-Form Health Survey - SF36 and Voice Handicap Index (VHI).

**Study design:**

Prospective transversal cohort study. **Method and Material**: Fifty senior women participated in this research, with ages between 60 and 87 years and mean age of 70.8 years old, randomly recruited. The participants of the study were submitted to two questionnaires: SF36 and VHI. The answers of both questionnaires were compared by Kruskall-Wallis test, verifying if there were significant differences among the variables. The test of Spearman was used to evaluate if there was correlation among the results of the variables of VHI and the results obtained in the parameter of SF36 for life quality.

**Results:**

We obtained values considered statistically significant in the correlations among physical domain of VHI and physical operation, physical pain and physical role in life of SF36.

**Conclusion:**

There was a significant and positive correlation among the results obtained in the parameters physical operation, vitality, general health, mental health, corporal pain and physical role in life of SF36. There were statistically significant and negative correlations among the total results obtained in SF36 and VHI.

## INTRODUCTION

Getting old is an expected phase in people's life. However, those that make it, end up suffering significant losses, such as changes to their social role, loss of relatives and friends, separation from occupational activities, and the onset of chronic diseases^1^, ^2^, ^3^.

Aging of the population has different characteristics depending on life conditions of each country and region. In general, the necessary conditions to reach a long life have improved all over the world, resulting in increased elderly population.

The growing number of elderly people make us wonder about the needs of this population, how it deals with daily situations, and specially, what is the psychosocial impact of aging in the routine and quality of life of elderly people^3^, ^4^, ^5^, ^6^, ^7^, ^8^, ^9^, ^10^.

The term quality of life is frequently used in the scientific context. The attention of governmental agencies to the elderly and the progression of medicine have contributed to it; thus, the elderly population has better healthcare conditions. However, this input does not necessarily mean that quality of life will also improve^12^, ^13^, ^14^.

One of the health conditions that may cause repercussions in quality of life is vocal conditions^6^, ^7^, ^8^.

Voice is a preponderant factor for communication and it reveals physical and psychological characteristics of the subject. In senescence, vocal changes may negatively interfere in the relation of subjects and in the social adjustment of elderly people^2^, ^6^, ^7^, ^8^, ^9^, ^10^.

We found literature reports about the specific characteristics of vocal physiology and anatomy of the elderly, but little has been said about the concern to learn about the impact of vocal conditions on quality of life in this part of the population^2^, ^3^, ^6^, ^8^, ^9^, ^10^, ^11^, ^15^.

In the present study, we decided to check the impact of voice on quality of life of elderly women using the Questionnaire Short-Form Health Survey - SF36 (15), adapted to Portuguese, to measure quality of life and the questionnaire Voice Handicap Index - VHI to describe the impact of voice on emotional, physical and social life of people^16^.

## MATERIAL AND METHOD

Fifty elderly women participated in the present study; they were aged 60 to 87 years, mean age of 70.8 years, resident in a city of 200,000 inhabitants in the rural area of the state of Sao Paulo, which were randomly recruited in the streets of the city. The inclusion factors were age equal or greater than 60 years, good mental and physical health status, non-smoker, no history of vocal or neurological disorders and chronic respiratory pathway diseases.

The sample comprised fifty consecutive volunteers that met the inclusion and exclusion criteria.

All subjects signed the informed consent term approved by the Research Ethics Committee, Pontifical Catholic University of Sao Paulo.

Participants were submitted to two questionnaires that they were supposed to read and answer by themselves in writing within one hour. All relevant questions concerning the questionnaires were answered by one of the authors.

Each questionnaire has been regularly used all over the world and data collection and checking methodologies have been dully validated. We assessed the following parameters: SF36 assessed general health, mental health, vitality, body pain, physical functioning, social functioning, emotional role in life, and physical role in life. Using VHI, we observed physical, emotional and functional aspects of voice.

SF36 is a questionnaire created by Ware (1993), and it was extensively referred and approved by many areas of Human Health, and it can be applied to anyone over the age of 14 years, and consists of 8 subcategories in the concept of health: physical functioning, social functioning, body pain, vitality, emotional and social roles in life, general and mental health status. All subcategories are assessed by different questions in which the subject has to choose between different answers offered by the questionnaire that will define a final numeric score (Table 1).

**Table 1:** Items assessed by different categories of questionnaire SF36.

Items	Categories
Vigorous activities	Physical functioning
Moderate activities	
Elevate, carry vegetables	
Climb up the stairs	
Climb up one step	
Bend down, kneel down	
Walk 1 km	
Walk many blocks	
Walk one block	
Take a shower, get dressed	
Reduces time	Physical Role
Manages less	
Limited to some types	
Has difficulties	
Pain severity	Body pain
Interfered by pain	
Appropriate general conditions	General health status
Frequently sick	
Just like before	
Getting worse	
Excellent health	

VHI was proposed by Jacobson, Grywaslki, Silbergleit, Benninger and Newman (1997) and describes the impact of voice and its affections to people's lives. The instrument is divided into three subcategories: physical, emotional and functional impact. All categories have ten questions to be answered and the final count depends on the answers chosen from a list of words: never, almost never, sometimes, almost always, always.

Thus, we managed to compare what the person presented as quality of life (SF36) and vocal conditions, considering the capabilities everyone had to perform tasks that depended on their voices.

We collected responses from both questionnaires and summed up the results to reach the total final score for both and the results were compared by non-parametric Kruskall-Wallis test, to check whether there were significant differences between the variables physical, emotional and social impact of VHI questionnaire and all subcategories of SF36. Non-parametric Spearman test was used to assess whether there was correlation between results of physical, social and emotional impact of VHI and results obtained in parameter SF36 of quality of life.

## RESULTS

Table 2 shows the counts obtained for each analyzed variable using SF36.

**Table 2:** Numeric results obtained for each variable of SF36.

subjects	FF	PF	D	variable SG	V	FS	PE	SM	Mean
1	95	75	80	75	95	100	100	96	89.50
2	55	0	74	57	60	100	100	56	62.75
3	85	25	62	87	50	75	0	60	55.50
4	70	100	41	72	55	100	100	76	76.75
5	45	50	84	67	100	100	66.7	100	64.08
6	80	50	74	42	80	75	100	80	98.12
7	90	100	41	72	85	100	100	84	84.00
8	95	100	100	97	85	87.5	100	96	95.68
9	55	100	74	42	90	100	100	96	81.50
10	90	50	74	87	85	87.5	66.7	92	79.02
11	90	100	100	100	85	87.5	100	92	83.06
12	100	100	84	100	95	100	100	100	98.00
13	80	75	64	100	100	75	100	96	85.00
14	85	75	41	80	90	62.5	100	60	71.68
15	85	75	72	97	70	100	33.3	96	79.78
16	35	0	72	50	80	100	33.3	64	50.53
17	40	75	51	80	50	100	100	92	76.62
18	75	100	84	85	75	100	100	76	85.62
19	65	50	72	97	65	75	100	80	75.50
20	60	0	41	40	65	62.5	0	48	38.31
21	50	75	51	60	55	87.5	100	56	66.80
22	75	75	62	52	75	100	66.7	88	74.21
23	90	100	74	82	75	75	100	56	81.50
24	95	100	74	92	65	87.5	100	48	82.68
25	55	75	41	97	75	100	0	60	62.87
26	80	50	22	80	65	50	66.7	100	64.21
27	100	100	84	52	50	87.5	100	72	80.68
28	65	75	40	67	75	87.5	66.7	88	70.52
29	80	100	84	75	75	50	33.3	60	69.70
30	85	100	62	77	75	100	100	100	87.37
31	95	100	100	87	90	100	100	88	95.00
32	35	25	0	85	35	25	33.3	40	34.78
33	90	75	72	92	95	100	100	80	88.00
34	100	100	84	55	80	100	100	88	88.37
35	50	50	41	45	70	100	100	80	67.00
36	40	50	42	45	55	62.5	0	56	43.81
37	100	100	100	95	50	100	100	100	93.12
38	40	100	41	82	85	62.5	0	84	61.81
39	90	0	32	82	65	75	100	72	64.50
40	75	75	42	82	90	100	100	92	82.00
41	95	100	100	90	40	100	100	88	89.12
42	60	100	10	45	70	87.5	100	68	67.56
43	75	100	74	87	80	87.5	100	84	85.93
44	50	100	74	72	90	87.5	100	92	83.18
45	100	100	100	100	95	100	100	96	98.87
46	55	100	74	72	65	50	0	84	62.50
47	80	100	51	67	80	87.5	100	72	79.68
48	30	0	10	40	40	62.5	33.3	28	30.47
49	100	25	10	97	95	87.5	0	84	62.31
50	100	100	100	100	95	100	100	100	99.37
Mean	74.2	73.0	67.72	75.62	73.6	58.5	66.0	78.88	74.97

FF - Physical functioning; PF - physical role in life; D - body pain; SG - general health; V - vitality; FS - social functioning; PE - emotional role in life; SM - mental health.

Table 3 shows counts for variables analyzed by VHI.

**Table 3:** Numeric results obtained for each variable of VHI.

subjects	variable			
	Physical	Emotional	Functional	Total
1	0	2	2	4
2	9	8	7	24
3	8	0	1	9
4	0	4	2	6
5	0	0	0	0
6	0	0	0	0
7	0	0	0	0
8	4	3	0	7
9	2	4	1	7
10	0	0	0	0
11	0	3	1	4
12	4	2	2	8
13	0	2	3	5
14	6	4	2	12
15	4	3	3	10
16	13	0	16	29
17	15	2	7	24
18	0	0	2	2
19	6	4	8	18
20	3	2	3	8
21	2	2	2	6
22	24	21	13	58
23	0	0	2	2
24	0	0	0	0
25	2	6	0	8
26	8	0	2	10
27	0	0	0	0
28	6	15	14	35
29	11	8	0	19
30	0	3	2	5
31	0	0	0	0
32	6	2	7	15
33	18	14	10	42
34	0	0	0	0
35	0	0	1	1
36	6	1	15	22
37	0	0	0	0
38	2	1	2	5
39	0	0	0	0
40	0	0	1	1
41	0	0	0	0
42	0	0	0	0
43	0	0	0	0
44	2	0	0	2
45	6	0	0	6
46	2	0	0	2
47	0	0	0	0
48	0	0	7	7
49	0	0	0	0
50	0	0	1	1
Mean	3.38	2.32	2.78	8.48

Statistical analysis of VHI consistency of results is shown in Table 4.

**Table 4:** p values for the comparative analysis of each VHI variable.

Pair of variables	Physical vs. Emotional	Physical vs. Functional	Emotional vs. Functional
p value	0.48 (N.S)	0.52 (N.S)	0.62 (N.S)

Statistical analysis of SF36 consistency of results is shown in Table 5.

**Table 5:** p and r values for correlation of subcategories of SF36 when there were statistically significant differences (Spearman test, p ≤ 0.05)

Subcategory pairs	p value	r value
Physical Functioning versus physical role in life	0.0033	0.407
Physical Functioning versus body pain	0.0013	0.514
Physical Functioning versus general health status	0.00062	0.535
Physical Functioning versus Vitality	0.011	0.353
Physical role in life versus body pain	0.00010	0.520
Physical Role in life versus Vitality	0.037	0.295
Physical Role in life versus Mental health status	0.019	0.3306
Body pain versus General health status	0.016	0.339
Body pain versus Vitality	0.021	0.324
Body pain versus Mental health status	0.019	0.428
General health status versus Vitality	0.005	0.3841
General health status versus Mental health status	0.0064	0.3805
Vitality versus Mental health status	0.000001	0.625

The statistical result of the comparative analysis between SF36 and VHI is shown in Table 6.

**Table 6:** p and r values for the correlation between subcategories of SF36 and VHI categories (Spearman test, p ≤ 0.05)

SubcategoriesVHI	SF36	r values	p values
Physical Functioning x physical		-0.325	0.020
Physical x physical Role in life		-0.315	0.025
Functional x physical Functioning		-0.044	0.0010
Functional Role x physical in life		-0.453	0.0009
Functional x Body pain		-0.35	0.010

## DISCUSSION

There are few studies relative to the impact of vocal condition of the elderly on quality of life. Trying to better understand this aspect, we used an instrument to measure quality of life as an expression of general health status (SF36) and an instrument to describe the psychosocial and functional impact of voice on subjects' lives (VHI). SF36 is considered an appropriate scale to measure quality of life, as suggested by Cella & Tulsky (1990), because it assesses more than one aspect related with quality of life and measures daily activities. Lyons et al. (1994) stated that SF36 is an instrument that can be used to interview elderly people with different mental status^17^, ^18^, ^19^, ^20^, ^21^, ^22^. The instrument used to measure vocal impairment (VHI), according to Jacobson et al. (1997), is designed to measure the effects of social and psychological damage of vocal affections. This opinion is shared by other authors such as Rosen & Murry (2000) and Courey et al. (2000).

As to VHI questionnaire, 80% of the subjects presented total count between 0 and 12 points, out of a total of 120 points (Table 2). These data indicate that most of the subjects presented low level of vocal impairment, in other words, they considered their voices appropriate to their daily activities. This finding is similar to that of Rosen & Murry (2000) when they studied the use of VHI in a group of singers and non-singers.

The responses of participants to many different subcategories of VHI were submitted to statistical analysis using Kruskall-Wallis test and there were no statistically significant differences between them (p = 0.48 physical and emotional; p = 0.52 physical and functional; p = 0.62 emotional and functional) (Table 4), indicating that responses given to each parameter presented similar behavior. Statistical analysis also demonstrated strong correlation between subcategories, meaning that functional, physical and emotional impairment progressed similarly (Table 2).

As to domains of questionnaire SF36, we obtained high values for means of each domain (Table 1). Each domain of SF36 questionnaire allows scores from 0 to 100. The higher the score, the better the health condition. To learn whether there was correlation between questionnaire variables, Spearman statistical correlation test was applied and did not show significant differences between them.

Spearman correlation test was applied and we established correlation between each one of the assessed parameters with SF36 questionnaire. We found strong positive correlation among the variables. This fact suggests that body pain, general health conditions and well-being feelings, depression, anxiety, and emotional control make the elderly people stop to do things or to have difficulty to finish the work because they feel that their health status limits their professional and social activities.

In order to learn whether there was any correlation between questionnaires SF36 and VHI, we applied Spearman correlation test again but this time among variables from each of the forms. We reached statistically significant values in correlations between physical domain of VHI and physical functioning, body pain and physical role in life of SF36 questionnaire.

The correlation between physical domain and physical functioning (p = 0.020 and r = -0.325) may mean that the change in vocal quality, difficulty to talk and vocal instability, which are present in aging, may cause limitations in the conduction of daily physical tasks such as: going shopping, walking, taking a shower, and practicing sports. We observed that even though they did not have use of voice in physical activities assessed by the physical functioning scale, these two aspects - physical domain and functioning physical aspect - walked hand-in-hand.

The results that presented high physical impairment, without interference in specific activities, could have indicated that subjects were well adapted, regardless of vocal characteristics present in aging. However, abnormalities to vocal quality, effort when speaking, instability and vocal fatigue may be some of the factors that lead the elderly population to seek for healthcare services. Based on it, we can deduct that when working with elderly people, therapeutic approach should focus on preventive aspects and increment of vocal efficiency. To minimize the effects of presbiphonics, specific vocal training should be addressed to vocal quality and speech and articulation dynamics. Moreover, Morrison & Rammage (1994) and Sataloff; Rosen; Hawkshaw; Spiegel (1997) stated that the effects of aging in voice can be less evident in the presence of good physical conditions.

Once established the correlations and consistencies between the various parameters in each questionnaire, we investigated the correlation between total counts of VHI and SF36. Statistical results did not show statistically significant differences. However, the analysis of values obtained for each subject showed that only three of them had high total count of VHI. Women with extreme impairment were excluded from the analysis and covariate analysis was conducted in the rest of the group, showing strongly negative covariation (x = -48.78), or in other words, the higher the vocal impairment, the worse the quality of life. Spearman correlation test was also applied and we reached correlation between total count of SF36 and VHI as r = -0.45 and p = 0.049, showing that there was significant negative correlation between quality of life and vocal impairment, confirming the results described above.

These results are in accordance with the study by Benninger et al. (1998), conducted to assess the correlation between SF36 domains and VHI categories in patients with vocal affections, in which they detected that vocal affections had significant impact on quality of life of subjects. Similarly, the study by Ferreira et al. showed impact on quality of life and lifestyle of patients (1994).

## CONCLUSION

•Physical, emotional and social parameters of VHI were coherent and correlated;

•There was statistically significant positive difference between results obtained in physical functioning, vitality, general health status, mental health, body pain and physical role in life from SF36;

•There was statistically significant and negative difference between total result obtained in SF36 and VHI. Thus, vocal conditions may significantly interfere in quality of life of women aged over 60.
